# Access to Guideline-Concordant Oncology Genomic Testing: A Qualitative Study of Black Cancer Patients and Oncology Providers

**DOI:** 10.3390/curroncol33070413

**Published:** 2026-07-10

**Authors:** Andrea Thoumi, Yadurshini Raveendran, Laura Fish, M. J. Gathings, Emily Rosario, Shaun R. Jones, Hayden B. Bosworth, Linda Sutton, John H. Strickler, Tomi Akinyemiju

**Affiliations:** 1 Department of Population Health Sciences, Duke University School of Medicine, Durham, NC 27701, USA; 2 Duke Cancer Institute, Duke University School of Medicine, Durham, NC 27710, USA; 3 Global Pediatric Medicine, St. Jude Children’s Research Hospital, Memphis, TN 38105, USA; 4 ETR Services, Raleigh, NC 27705, USA; 5 UNC Lineberger Comprehensive Cancer Center, Chapel Hill, NC 27599, USA; 6 Department of Epidemiology, Gillings School of Global Public Health at University of North Carolina (UNC) at Chapel Hill, Chapel Hill, NC 27599, USA; 7 Center for Innovation to Accelerate Discovery and Practice Transformation (ADAPT), Durham Veterans Affairs Medical Center, Durham, NC 27701, USA

**Keywords:** genomic testing, cancer disparities, black patients

## Abstract

Genomic testing is a key component of precision oncology; however, Black patients receive genomic testing at lower rates. The purpose of this qualitative study was to identify individual and health system drivers of genomic testing disparities at a National Cancer Institute-designated comprehensive cancer center. We conducted interviews with 15 oncology providers and 11 Black cancer patients between September 2023 and October 2024. These patients were eligible for genomic testing based on National Comprehensive Cancer Network (NCCN) guidelines, being diagnosed within last 10 years (2014–2023), at least 18 years old, and English-speaking. Providers included oncologists and oncology patient navigators. Topics included motivators, barriers, and knowledge of genomic testing and factors influencing decision-making. The Penchansky and Thomas theoretical framework of healthcare access (e.g., availability, accessibility, accommodation, affordability, and acceptability) guided thematic analysis. Among patients eligible for genomic testing, most participants (*n* = 7) received genomic testing as part of their cancer treatment based on EMRs, however many patients (*n* = 7) could not recall discussing genomic testing with their oncologist. Most patients and all providers highlighted affordability as a challenge: patients were concerned about unexpected costs associated with testing, while providers were concerned about costs of matched molecular targeted therapy. Both patients and providers highlighted patient-centered communication to mitigate mistrust and promote patient engagement in care. Despite limited awareness, Black patients view genomic testing positively. Addressing multiple dimensions of access is key to improving system-level processes and ensuring that more patients benefit from lifesaving targeted therapy.

## Simple Summary

Cancer treatment has become increasingly personalized, with genomic testing used to identify specific features of a tumor that can guide treatment decisions. However, Black patients are less likely to receive genomic testing, contributing to disparities in cancer outcomes. In this study, we interviewed 15 oncology providers and 11 Black cancer patients at a major cancer center to understand why these disparities exist. We found that while most patients who received genomic testing viewed it positively, many patients could not recall ever discussing testing with their doctor. Both patients and providers identified cost as a significant barrier. Patients worried about unexpected out-of-pocket expenses and providers expressed concern about the cost of follow-up treatments. Trust and communication also emerged as critical factors. These findings suggest that improving awareness, strengthening patient–provider communication, and addressing financial barriers are essential steps toward ensuring that Black patients have equal access to potentially lifesaving personalized cancer treatments.

## 1. Introduction

In the United States (US), an estimated two million people will be diagnosed with and over half a million people will die of cancer in 2025 [1]. While disparities in survival rates across all cancers have narrowed over time, non-Hispanic-Black (Black) patients continue to experience higher mortality rates compared to non-Hispanic White (White) patients [1,2,3]. For example, mortality rate ratios across common cancers include 37% higher for breast cancer, 26–45% higher for colorectal cancer, 13% higher for lung cancer among males, and 108% higher for prostate cancer in Black compared to White patients [4]. Social determinants of health (SDOH) impact risk factors that are associated with cancer mortality including access to screening, early diagnosis, and receipt of timely and quality guideline-concordant care [5,6,7,8].

An important and growing component of guideline-concordant oncology treatment is the use of genomic testing to identify specific molecular alterations that may inform therapeutic strategy—an approach that has revolutionized precision oncology across many cancer types, extending survival and improving quality of life [9,10,11,12,13]. Genomic testing involves tumor profiling, molecular testing, or next generation sequencing (NGS) and analyzes patient samples for genetic mutations, alterations or expression patterns to inform disease classification, prognosis or highlight actionable mutations to target with specific therapies [10,14,15]. The American Society for Clinical Oncology (ASCO) recommends genomic testing for patients diagnosed with metastatic cancers or tumors known to exhibit at least one biomarker with an approved therapy [10,16]. The National Comprehensive Cancer Network (NCCN) guideline, which guides the use of genomic testing in oncology, also considers genomic testing a measure of high-quality care [17,18,19,20]. For instance, patients with non-small cell lung cancer identified as having anaplastic lymphoma kinase (ALK) rearrangement based on genomic testing could be prescribed ALK-targeted therapy, a therapeutic approach that is associated with improved survival compared with standard chemotherapy [13,18].

Despite the widespread recommendation of genomic testing and targeted therapies as guideline-concordant care, racial and ethnic disparities in genomic testing have been widely reported in many cancers [21,22,23,24,25]. For example, a recent study on metastatic prostate and urothelial cancers reported Black and Latino patients were 25% and 30% less likely than white patients to receive NGS testing, respectively [22]. Furthermore, studies on gene recurrence scores (RS) in breast cancer have found American Indian women are 14% less likely to receive RS compared to White women and Black women experience 11% lower prevalence of RS compared to non-Black women [24,26]. Additionally, a recent systematic review assessing genomic testing in lung cancer treatment found most studies (11 of 18) reported disparities in the use of genomic testing by race and ethnicity [21]. Research highlights SDOH, such as, insurance type, income status, and education attainment, contribute to lower likelihood of receiving testing or knowledge of genomic testing [22,27]. One study found cancer patients enrolled in Medicaid or low-income status were 47% and 26% less likely to receive genomic testing than patients on commercial insurance or high-income status [22]. While the role of SDOH on disparities in genomic testing is well-established, few studies have qualitatively assessed experiences related to accessing genomic testing among Black cancer patients [28].

The purpose of this study was to identify motivators and barriers to genomic testing among Black patients eligible for genomic testing due to a cancer diagnosis in breast, colorectal, lung, and prostate and oncology providers in a comprehensive cancer center setting. Further, we aimed to contextualize these experiences using the Penchansky and Thomas framework, which defines access through five dimensions: accessibility, affordability, availability, acceptability, and accommodation [29]. Previously, studies have deployed this framework to clarify cancer access disparities in quantitative analyses [30,31,32]. In addition, other studies have utilized the framework to qualitatively assess barriers related to mammography screening, inform a systemic review of access barriers related to breast cancer reconstruction surgery, and a mixed-methods study on access to radiation therapy [33,34,35]. To our knowledge, no study has utilized this framework to evaluate motivators and barriers among Black cancer patients accessing genomic testing. Insights from this study may inform interventions to improve utilization of genomic testing and targeted therapies among Black patients that continue to experience disparate cancer mortality rates.

## 2. Methods

### 2.1. Study Design and Recruitment

This study utilized a qualitative study design to characterize experiences of Black cancer patients supplemented by interviews with oncology providers through natural inquiry [36]. Patients and oncology providers were recruited from a large US comprehensive cancer center—the Duke University Cancer Institute (DCI). We identified eligible patients using electronic medical records (EMRs). Patient inclusion criteria were: Black race; diagnosed with breast, colorectal, lung, or prostate cancer [17,18,19,20]; eligible for genomic testing based on NCCN guidelines; diagnosed within the last 10 years (2014–2023); at least 18 years old; and English-speaking. Research team members trained in community-engaged approaches and qualitative methods contacted 30 patients by phone, providing information about the study and inviting patients to participate. Interested patients completed informed consent on the recruitment call and again at interview. Each participant received a $50 gift card as compensation. Inclusion criteria for providers were: DCI oncologists or oncology patient navigators with an active affiliation at DCI. Providers did not need to be involved in the care of participating patients.

### 2.2. Data Collection

We conducted one-to-one patient and provider interviews to assess knowledge of and factors that facilitate or hinder decision-making and utilization of genomic testing (defined as inclusive of NGS and targeted panels). We also conducted a focus group with a group of providers to accommodate scheduling conflicts. The discussion guide for patients included topics relating to knowledge, perception and willingness to utilize genomic testing, while the discussion guide for providers included topics such as familiarity with NCCN guidelines for identifying and referring eligible patients for genomic testing, and patient, clinic or institutional factors influencing testing. The provider interviews and focus group were conducted in September 2023 and patient interviews were conducted between February and October 2024. All discussions occurred on Zoom and were 1 h, audio-recorded, and transcribed. Two team members facilitated each session.

### 2.3. Analysis

Two independent coders used NVivo 12 software for data analysis. First, coders familiarized themselves with the transcripts. Then, coders reviewed the transcripts for knowledge, health communication, and health literacy related to genomic testing. To ensure a structured and systematic approach, the Penchansky and Thomas’ [29] theoretical framework of healthcare access guided the development of an a priori codebook and qualitative analysis. We defined each HCA dimension as the following: accessibility (e.g., distance to healthcare service), affordability (e.g., price, willingness, and ability to pay), availability (e.g., type, volume, and quality of care), acceptability (e.g., patient satisfaction with interactions with provider including trust), and accommodation (e.g., organization of healthcare services that account for patient needs and experiences) [37]. We incorporated additional codes that surfaced, allowing for a flexible and responsive analysis to emergent themes. An inductive and deductive approach resulted in final codes that focused on patient cancer journeys, awareness and perceptions of genomic testing, and healthcare access. Coders met regularly to compare coding decisions, share interpretations, and resolve discrepancies in consensus meetings. Coders reached consensus on all coding decisions, ensuring that the final coding was both accurate and reflective of the interview data, thus enhancing coding reliability and consistency.

### 2.4. Ethics and Consent Statement

This study was reviewed and approved by the Duke Health Institutional Review Board under Pro00112183. All participants provided informed consent for inclusion in the study.

## 3. Results

A total of 11 Black cancer patients aged 26 to 78 participated in the study. Participants included five men and six women diagnosed with the following cancers within the last 10 years (2014–2023): colorectal (*n* = 3), lung (*n* = 3), prostate (*n* = 3), and breast (*n* = 2). As our primary goal was to assess the racialized experiences of Black cancer patients navigating genomic testing, this sample size is consistent with established guidance for homogenous populations [38]. Of the 11 patients interviewed, seven patients had received genomic testing, and four patients had not received genomic testing at the time of the interview based on EMRs. A total of five providers participated in interviews: breast oncology (*n* = 1), breast surgeon (*n* = 1), medical oncology (*n* = 1), and oncology patient navigator (*n* = 2). In addition, genitourinary oncologists (*n* = 10) participated in a focus group.

### 3.1. Awareness and Interest of Genomic Testing Among Patients

Patients described their cancer treatment journey, including knowledge of and experience with genomic testing. Some patients were in remission while others were still receiving treatment at the time of the study. Most patients (*n* = 7, 64%) reported they did not recall discussing genomic testing with their care team. Two patients stated they heard about genomic testing from a friend or social media.

*Another friend of mine, she actually utilized genome test, genome, I don’t think I said that correctly, for her treatment. What she talked about was the technology around trying to decide, like you said, decide on the right path for treatment and if in fact they would take that. I think I got this right. That they can even use the sample to even see if your body will react a certain way.—Patient*

*[When asked about genomic testing]…I don’t think we had another conversation about it so I don’t know.—Patient*

*When you say generic testing, see that’s where I’m, that’s where you’re losing me…..What is that?—Patient*

After the interviewer explained the purpose and benefits of genomic testing, most patients (*n* = 7, 64%) shared they would be willing to receive genomic testing.

*I’m interested in it. It seems like it’s a very viable option that should be offered. I would like to, knowing what I know now.—Patient*

*What excites me would be if they could find the medicine that would actually shrink the, my tumors… as long as it does more good than harm, I would probably still try it… I would decide to do it but that would also depend on if I got approved for like the financial assistance.—Patient*

*I actually wished that had happened for me. Like I said, maybe it did, I just don’t remember. The unfortunate part about being on chemo is that chemo brain is real.—Patient*

Only two patients self-reported knowing that they had received genomic testing during their cancer journey. Of these patients, one noted they were initially hesitant to receiving testing and only agreed to the test after the care team explained the benefits.

*Actually they said, initially they reported that genomic testing was primarily of interest for those who had children to see if things would genetically pass onto the children. Since we weren’t going to have children we said no, we’re not really interested in that. But then they said ‘well, we want to see if your cancer will respond to some things versus other things,’ so then we said yes and that’s basically all there was to it as far as I know.—Patient*

### 3.2. Prescribing Genomic Testing by Providers

Providers were asked about their perception and referral patterns for genomic testing, including factors that influence their decision to order genomic tests for patients. Most providers reported routinely ordering genomic tests in accordance with clinical guidelines; only one provider stated that they neither order nor use genomic testing for their patients, which may be due to variation in genomic testing relevance by specialty or role.

*Both my nurses on both sides know how to order it. So we definitely streamlined that process to make it like as accessible for my team or in our team as possible.—Provider*

*I don’t order it but I have lots of patients who get it and so, you know, when patients get things that have to do with stuff that I’m treating I do have to have some understanding of it. So, in my way it’s something I have to be aware of, have a little bit of understanding of but I don’t personally order those tests. I don’t personally manage the results of those tests but certainly my patients will get those results and I’ll be the first person they see. ‘Dr. (Name), what does this mean?’ So, I do have to have some understanding of it.—Provider*

*I think those things you need is worrying about, we, we do see a lot of second opinions or it’s often times they just will come to me and I’ll be like you haven’t had the testing done. I’ll order the testing but then they return to South Carolina or wherever they were living to see their local oncologist and making sure that those results are relayed to the treating oncologist can be challenging.—Provider*

Providers identified several opportunities to improve patient–provider informed decision-making, including improved availability of education materials for patients and patient knowledge assessments of genomic testing in advance of a medical appointment. They also suggested ways to improve sharing results with other providers and patients, such as through increased documentation in EMR and training for providers.

*We don’t have in our notes any area that we document this consistently and you know, the different tests or the different areas of the EMR and EPIC whatnot, so, you know, I think we have room for improvement in how we systematically ensure that this is done.—Provider*

*I think that what I struggle with is the patients who don’t know what they don’t know is when you ask them, they kind of just say, ‘Well, whatever you normally say.’ Or, ‘What am I forgetting to ask?’ and that’s hard. It’s hard for me to guess what you don’t know… I think that it would be helpful maybe if maybe there was some kind of assessment the patients did either right before the visit or right after that would kind of give me a little bit of that information going into the conversation and then maybe that could help guide my conversation, maybe something like that would help.—Provider*

### 3.3. Motivating Factors and Barriers to Genomic Testing

Most patients (*n* = 8, 73%) and all providers identified affordability as a barrier to genomic testing, specifically based on insurance coverage and availability of financial support to cover associated costs. Many patients (*n* = 7, 64%) also highlighted that better understanding of the risks versus rewards associated with genomic testing would have influenced their decision-making process. Several patients shared that they would be comfortable with genomic testing since it uses previously removed tissue and would not require a separate procedure.

*If insurance would cover it and it and it’s more options beside chemo I mean I ain’t got, like I said, I ain’t got no problem with the test.—Patient*

*It seemed really effective. They had good results out of it. Oh, I know what it was. The insurance wouldn’t cover it…So, that’s kind of why, one of the main reasons I ruled it out.—Patient*

*Well, if the insurance don’t pay for, I don’t even want to look into no other options. I won’t be able to afford it.—Patient*

*The time, the money, the cost, how much would I have to travel because I live out of the state from (State).—Patient*

*Um, that would be my only concern, just by the financial assistance and the benefit of doing it, does it actually, will it actually help me in the long run.—Patient*

*I think it, that would be low on the, on the decision tree as far as not electing to do it, it’s just basically, when you, when you speak of cost and, and financing, you know, whether it’s through out of pocket or through insurance or whatever, for Black people that’s, that’s a major, major, major decision and that is, that along with the lack of information. I would decide to do it but that would also depend on if I got approved for like the financial assistance.—Provider*

Aligned with the acceptability domain of healthcare access, patients also shared that historically grounded distrust of healthcare systems, fear, lack of access to information, and provider bias are all potential drivers of genomic testing disparities in Black patients. Furthermore, potential implicit bias in provider assumptions about patient receptivity to genomic testing may affect whether genomic testing is offered to Black patients in the first place.

*I think one of the first questions would be like the accuracy of it as it relates to the treatment, knowing me, I would probably have asked how many times have you done this?—Patient*

*And as you pointed out, there is a whole bunch of history where, you know, the best interest of people of color was not taken into account.—Patient*

*And that’s just part of our culture and it’s kind of, it goes generation to generation and it’s just, you know, it’s just part of that lack of trust.—Patient*

*Well, I think that sometimes certain conversations need to be had by someone that look like the demographics you’re trying to… and I think that’s important to have someone that can speak to that and share with people that look like them because then they are a little bit more trusting with it.—Provider*

*You know, where the patient’s receiving their care. Is it rural community practices versus an academic practice and there’s disparity in just in which patients walk in our door, their socioeconomic status, their education levels, whether they’re asking for the test, the type of provider they’re seeing. So I do think there’s disparity right from the beginning of those patients coming to (Hospital).—Provider*

*Maybe they just, the provider, not provider in the insurance carrier provider, I’m saying the provider, the caretaker, i.e., doctor, nurse practitioner or the navigator. Social worker, anyone, that they may not share that with that patient. Because they have maybe already assumed that that patient would not do it [crosstalk]. Without even having a conversation with the patient. They’ve already you know, have some type of judgement about that particular patient. So I think it may be a you know, lack of knowledge.—Provider*

Providers generally indicated that they would be more likely to order or refer testing based on whether the results would influence the patient’s treatment. However, a provider stated the lack of diversity in trials could be contributing to current disparities in Black patients’ use of genomic testing and next generation sequencing.

*We use it only right now in our stage 4 patients. So patients with metastatic disease. For the breast cancer space it definitely depends a lot on the subtype of breast cancer that they have. So for example, patients with triple negative breast cancer it’s very important because we already are very limited in what treatment options those patients have in terms of targeted therapies and so next generation sequencing or really PD-L1 testing dictates our first line of treatment for those patients and so at least in-house we already send PD-L1 testing but getting the rest of that next generation sequencing we usually set for those patients up front at their diagnosis or like right, if they progress after their first treatment. We should be saying that on all patients.—Provider*

*I mean I could state with prostate cancer since that’s the most common scenario that we have in GU oncology and where tumor testing is very strongly recommended by national and international guidelines and I would say that most of our patients have tumor testing done.—Provider*

*You know what I mean and I can see it in other types of testing that sometimes people would do that same thing for whatever reason and so I think that, that could play into it too. Again, it’s like these assumptions that you have and so you say things in a different way and kind of gloss over things that maybe you shouldn’t cause maybe that is important to the patient if you explain to them a little bit more about what it was you wanted to do or might do and again, what those risks and benefits area kind of in light of whatever their values and preferences are.—Provider*

*I think, I think one problem is getting the test. I think another problem is that we don’t do the testing enough in diverse populations and so I think sometimes we have a harder time understanding the results in populations because, you know, even in germline testing we know that certain mutations mean a certain thing but all of that was found in a purely like European derived cohort and so like when somebody of African descent has that does it mean the same thing? I don’t know. Maybe, maybe not. And so I think that, you know, some of it is that providers might not feel as comfortable applying knowledge in areas where it wasn’t actually validated or developed and I think that maybe there’s some discomfort there that way. I think maybe providers put some assumptions into things that they shouldn’t. They say, oh well, I’ve known them for five seconds. They wouldn’t care what their germline genetic test results or whatever the test result might be. So no need to order it. And I think that they maybe skip over the conversation or they minimize it. I’m guilty of doing that, not necessarily based on race but I do it a little bit based on age.—Provider*

Both patients and providers offered strategies to facilitate greater use of genomic testing in Black patients, including increasing financial assistance, building patient trust and confidence in providers, and offering multimedia informational materials. Patients recommended using well-organized informational websites and outreach initiatives to reduce lack of information. Providers also recommended making point of care testing standard practice.

*I wish you could get this information out to us somehow and I’m trying to figure out, like I said, maybe through the medical oncologist and promote the team approach in other hospitals.—Patient*

*I think pamphlets. I think pamphlets would be great. Any kind of, even if they do commercials on the, even this company, just to get the word out cause I didn’t even know this existed until you, until you all called.—Patient*

*I personally think we should do more point of care testing. I think we lose a lot of patients in that pipeline when I talk to them about it and they’re like okay, yeah, that sounds interesting and then by the time they go the next appointment or they miss it, I just think we lose patients that probably would benefit from it when we don’t offer, you know, at the moment testing, point of care testing.—Provider*

*All the data and all that. For NGS testing in general, I really will just refer them to the company site that I’m going to send the testing from cause. I do feel like sometimes these companies have a lot of good patient education material on their sites.—Provider*

## 4. Discussion

In a qualitative study of Black cancer patients and oncology providers at a large US comprehensive cancer center, we investigated motivators and barriers to genomic testing. We analyzed and synthesized data from patient and provider interviews to identify key themes. Overall, study participants viewed genomic testing positively for its potential to identify relevant genetic mutations to inform treatment decisions. Our study revealed a key gap between patient awareness of receiving genomic testing and clinical data. Among the 11 patients interviewed, seven had received genomic testing while four had not; however, seven patients reported not knowing if genomic testing was part of their treatment plan. While aspects of all five HCA dimensions were discussed, patients and providers consistently noted the role of affordability, acceptability, and availability in genomic testing. Our study also highlighted key patient- and system-level opportunities to reduce disparities by improving financial incentives, provider training, and patient education material. See Table 1 summarizing key findings.

First, affordability, including whether and by how much insurance covered the testing costs, was a key factor in patients’ stated ability to receive the test, and in providers’ willingness to prescribe the test. Providers also highlighted that patients who are low-income or live in rural settings may not be offered genomic testing due to clinical setting or type of health insurance. Second, participants emphasized how medical mistrust and implicit bias (i.e., acceptability) can result in disparities in who is offered, and in patients’ willingness to accept, genomic testing. Similarly, participants highlighted that improved patient communication regarding the benefits of testing for treatment was an important factor to decision-making. Finally, participants emphasized how information sharing through educational materials can improve quality of care provided to all patients (e.g., availability). Although use of Penchansky and Thomas’s framework is relatively underutilized in cancer, these findings are consistent with a prior qualitative study examining access to mammography screening among Black women that also found patient-level and system-level barriers across all dimensions [33].

Prior research supports that while most patients are willing to utilize access genomic testing, cost and limited awareness of testing benefits are key barriers [28,39,40,41]. For example, a recent scoping review found that patients are concerned about insurance coverage and out-of-pocket payments (co-pays or self-pay) to cover the often high-cost of genomic testing [28,42]. This is consistent with our findings that affordability was an issue for both patients and providers. In addition, acceptability—quality of patient–provider interaction—was also a commonly described driver of genomic testing disparities. The limited research that focused on Black patients highlights that medical mistrust can act as a barrier to genomic testing, but can be mitigated through improved patient-centered communication to address patient concerns and increase awareness [43,44]. This is consistent with our study findings that patients raised concerns about medical mistrust, warranted fear, and implicit bias that may reduce uptake and exacerbate differences in genomic testing rates among Black patients. The historical reality that many academic medical centers in the US were segregated until 1965 continues to reverberate today [45]. Patient navigators that understand community concerns could improve trust and increase awareness of testing benefits while offering financial assistance to cover the costs of testing and molecular targeted therapies.

Our study also identified patient-level strategies that can improve awareness and uptake of testing. For instance, participants recommended that genomic testing companies and pharmaceutical companies that develop targeted therapies can create engaging, culturally relevant patient-facing educational materials to increase awareness, knowledge, and acceptability among patients. Such strategies can be successful if they consider patient context, such as perceived discrimination and implicit bias, that influence patient decision-making. For instance, tailoring patient education materials by using community-engaged principles can help incorporate lived experiences and help overcome patient concerns that are not explicitly addressed in standardized educational material [46]. In addition, patient education may need to distinguish between germline (e.g., BRCA testing) and somatic testing (e.g., tumor testing that informs biomarker targeted therapies) as patient consideration for each test might vary. We also identified a potential patient–provider communication gap as indicated by differences between self-reported and EMR-reported receipt of genomic testing data. While this gap may be partially explained by recall bias, short clinical encounter times may also result in limited time for providers to explain every test in detail. As a result, improved provider education on how to communicate the option and utility of genomic testing to patients may be needed.

Providers also suggested system-level initiatives to address affordability, such as securing financial assistance directly from companies providing the genomic testing for patients. Health system leaders could also proactively account for how different insurance types (e.g., Medicaid, ACA marketplace, employer-based) affect patients’ ability to pay. While several insurers in North Carolina (NC), including Medicaid and Medicare, have included coverage for genetic testing to identify inherited DNA (e.g., BRCA gene) that increases an individual’s cancer risk, until recently NC lacked a standard coverage policy for genomic testing. In 2025, NC passed state legislation (e.g., HB 567) to cover biomarker testing, a form of genomic testing used to identify cell mutations and inform cancer treatment, effective as of 1 October 2025 [47]. Given this study’s findings, an actionable next step is to increase health system awareness of the expanded coverage and develop financial processes to ensure all patients diagnosed with cancer and eligible for genomic testing are routinely offered free or low-cost genomic testing. Moreover, our study identified how gaps in interoperability can delay discussions and affect patient experience. For example, while some providers reported notetaking in EMR, there is no standard practice for documenting these tests or results, with relevant information often buried within EMR test and challenging to track. Additional challenges related to transferring results from comprehensive or academic cancer centers where genomic testing might be more routine, to community-based or rural providers, which may delay or hinder appropriate care for patients seeking care outside of academic medical centers. Therefore, efforts to standardize data reporting and enhance interoperability to streamline information sharing will likely make a difference in promoting uptake of genomic testing in line with clinical guidelines and patient needs and preferences.

Our study had several limitations. First, our sample size of providers was limited. Nevertheless, including patient and provider perspectives allowed for a more comprehensive assessment of access concerns. Second, while patient characteristics demonstrated balanced distribution across gender and cancer type, only two patient participants self-reported having received genomic testing. Thus, our results may have missed additional barriers that patients experienced during their treatment journey. In addition, non-Hispanic White, Hispanic, and American Indian patients may have additional access barriers not directly captured in this study. However, given the well-established disparities in testing, treatment quality and cancer mortality rates experienced by Black patients, insights and strategies to address these gaps will likely be beneficial across race/ethnic and socio-economic groups. Third, some institutional level mechanisms, such as funding for genomic testing, may have been unreported due to lack of awareness or knowledge by all participants. Furthermore, this study describes insights from a single comprehensive cancer center in one state, and so may not fully reflect the range of experiences or barriers at other institutions or comprehensive cancer centers. Future research that explores additional patient experiences, including in other race/ethnic groups, settings and across cancer types, type of diagnosis, and stage of treatment will be important next steps. In addition, health systems can consider implementing community-engaged approaches to identify contextually relevant modes of communication to increase education related to the benefits of testing. Additional future research directions may include expanding assessments to other testing types such as germline testing and evaluate treatment decisions. Finally, the field may benefit from a national survey conducted by the American Society of Clinical Oncology (ASCO) to systematically characterize access barriers including insurer coverage and patient copayments by state and type.

## 5. Conclusions

We identified key healthcare access dimensions (e.g., affordability, acceptability, availability, and accommodation) that may explain disparities in genomic testing among Black cancer patients. Building on these findings, we are evaluating high-intensity navigation for patients with metastatic breast cancer at-risk of non-guideline-concordant care including genomic testing. Specifically, we are assessing disparities in guideline-concordance by race and ethnicity, neighborhood area deprivation, subtype and over time. In addition to addressing financial barriers, interventions should address patient lack of awareness and medical mistrust, and improve system-level processes and interoperability to ensure access to timely, guideline-concordant genomic testing.

## Figures and Tables

**Table 1:** Key summary findings.

HCA Dimension	Key Finding	Identified Strategies
**Affordability** Price, willingness, and ability to pay for healthcare service (e.g., income, insurance, uninsurance, poverty)	Insurance coverage and out-of-pocket costs shaped both patients’ ability to pay for testing and providers’ willingness to prescribe it	Secure direct financial assistance partnerships; account for insurance type (Medicaid, ACA, employer-based) in access planning; leverage NC HB 567 (biomarker testing coverage, effective 1 October 2025) to ensure routine free/low-cost testing for eligible patients
**Availability** Type, volume, and quality of care (e.g., number of providers, hospitals, specialists, hospital teaching status, quality metrics)	Most providers order genomic tests per clinical guidelines; providers identified gaps in education materials and EMR documentation/training; lack of diversity in clinical trials may contribute to disparities	Increase availability of patient education materials; implement pre-visit patient knowledge assessments; improve EMR documentation standards and provider training on result-sharing; advocate for more diverse trial enrollment
**Accessibility** Distance to healthcare service (e.g., travel time, mode of transportation, distance to hospital, rurality)	Patients living in rural areas may be less likely to receive testing due to clinical setting or insurance type; gaps in transferring results between providers delay or hinder care	Standardize data reporting and EMR interoperability to streamline transfer of results to community or rural providers
**Accommodation** Organization of healthcare services that account for patient needs and experiences (e.g., hospital bed size, wait times, access to support services)	Gap between patients recalling receiving genomic testing and discussing with care team; short encounter times may limit providers’ ability to fully explain testing to cancer patients	Build patient knowledge assessments into pre-visit workflows; restructure encounters and documentation practices so genomic testing discussions are consistently captured and communicated; expand outreach initiatives to reach patients outside clinical encounters
**Acceptability** Patient satisfaction with interactions with healthcare service providers (e.g., trust, comfort, race concordance, reputation, transportation, time)	Historically grounded medical mistrust, fear, lack of access to information, and provider bias affect who is offered testing and patients’ willingness to accept it	Deploy patient navigators to build trust and address community-specific concerns; provider training to reduce implicit bias and improve patient-centered, benefit-focused communication

## Data Availability

The data that support the findings of this study are available in the supporting quotations in this article. Requests for additional information should be directed to the corresponding author.
